# Effect of Delayed Peripheral Nerve Repair on Nerve Regeneration, Schwann Cell Function and Target Muscle Recovery

**DOI:** 10.1371/journal.pone.0056484

**Published:** 2013-02-07

**Authors:** Samuel Jonsson, Rebecca Wiberg, Aleksandra M. McGrath, Lev N. Novikov, Mikael Wiberg, Liudmila N. Novikova, Paul J. Kingham

**Affiliations:** 1 Department of Integrative Medical Biology, Section of Anatomy, Umeå University, Umeå, Sweden; 2 Department of Surgical & Perioperative Science, Section of Hand and Plastic Surgery, Umeå University, Umeå, Sweden; University of Edinburgh, United Kingdom

## Abstract

Despite advances in surgical techniques for peripheral nerve repair, functional restitution remains incomplete. The timing of surgery is one factor influencing the extent of recovery but it is not yet clearly defined how long a delay may be tolerated before repair becomes futile. In this study, rats underwent sciatic nerve transection before immediate (0) or 1, 3, or 6 months delayed repair with a nerve graft. Regeneration of spinal motoneurons, 13 weeks after nerve repair, was assessed using retrograde labeling. Nerve tissue was also collected from the proximal and distal stumps and from the nerve graft, together with the medial gastrocnemius (MG) muscles. A dramatic decline in the number of regenerating motoneurons and myelinated axons in the distal nerve stump was observed in the 3- and 6-months delayed groups. After 3 months delay, the axonal number in the proximal stump increased 2–3 folds, accompanied by a smaller axonal area. RT-PCR of distal nerve segments revealed a decline in Schwann cells (SC) markers, most notably in the 3 and 6 month delayed repair samples. There was also a progressive increase in fibrosis and proteoglycan scar markers in the distal nerve with increased delayed repair time. The yield of SC isolated from the distal nerve segments progressively fell with increased delay in repair time but cultured SC from all groups proliferated at similar rates. MG muscle at 3- and 6-months delay repair showed a significant decline in weight (61% and 27% compared with contra-lateral side). Muscle fiber atrophy and changes to neuromuscular junctions were observed with increased delayed repair time suggestive of progressively impaired reinnervation. This study demonstrates that one of the main limiting factors for nerve regeneration after delayed repair is the distal stump. The critical time point after which the outcome of regeneration becomes too poor appears to be 3-months.

## Introduction

Peripheral nerve injuries (PNI) are common, with an annual incidence of 300 000 cases in Europe [1] and despite advances in surgical treatments the restoration of motor and sensory functions often remains incomplete. Since the mean age of the patient is between 18–44 years it is easy to understand the severity of these injuries. Apart from disabling the patient, the economical load on a personal level, as well as to the society in its entirety, is huge [2].

Several factors including the type of injury, age of patients and distance from the lesion site to the cell body can determine the severity of peripheral nerve injury and functional outcome following nerve repair. Distal peripheral nerve transection (e.g. in sciatic nerve) in newborn rats almost completely eliminates the corresponding spinal motoneurons within 2 weeks [3] while the same type of injury in adult animals has no effect on motoneuron survival [4], [5]. However, more proximal injuries to the spinal nerves [6], [7] and spinal roots [8] could result in significant degeneration in the ventral horn of the spinal cord. In contrast to motor cells, both neonatal and adult primary sensory neurons in the dorsal root ganglia undergo cell death even after distal peripheral nerve injury [5], [9]. The loss of sensory neurons is likely to contribute to the poor sensory recovery observed clinically after peripheral nerve lesions [10].

It has been shown that not only prolonged axotomy but also prolonged denervation could affect functional recovery following delayed nerve repair [11], [12]. The maintenance of muscle tissue is dependent on both electrical and neurotrophic stimulation which are both lost when the nerve is injured. This results in trophic, mechanical and molecular changes in the muscle. Following denervation the muscle undergoes progressive atrophy, the speed and force of contraction is reduced and there is often a conversion from a slow twitch phenotype towards fast twitch fibers [13], [14]. Muscle satellite (stem) cells are responsible for the growth, repair and regeneration of muscle tissue. Following nerve transection there is an initial increase in satellite cell proliferation, potentiating the capacity of regeneration in the newly denervated muscle. However, if the axotomy is prolonged the satellite cell number in the muscle tissue will dramatically decline. Thus, reinnervation is considered to be crucial for maintenance of satellite cell activity and number [15], [16].

There are however clinical cases where there is doubt whether the nerve injury is operable or not and whether it is worth to operate if there has been a time delay after injury. An example where this is an important consideration is the case of obstetrical brachial plexus injuries. Even with modern imaging techniques it is difficult to diagnose and to differentiate between preganglionic and postganglionic plexus injury and microsurgical reconstruction of the brachial plexus is often performed with 3–6 months delay [17] leading to poor recovery of sensation and reduced motor function in the affected limb [18]. Delay in surgical repair is also a well known factor significantly influencing the prognosis after median and ulnar nerve injuries [19]. Although it has been shown that early surgical intervention is beneficial for both motor and sensory recovery [20]–[22], there are no definitive clinical guidelines concerning the time delay when it is too late to perform a nerve repair. Peripheral nerve injury is often associated with significant loss of nervous tissue causing long nerve gaps and requiring nerve grafting to reconnect divided nerve stumps [10], [23]. The latter condition is yet another reason for the poor restoration of function following microsurgical repair of injured nerves [1].

The aim of this study is to use a clinically relevant experimental animal model of prolonged axotomy and denervation followed by delayed peripheral nerve grafting to determine when the regenerative capacity of the injured neurons becomes unacceptably low when repair is delayed and identify the mechanisms mediating poor axonal regeneration and muscle recovery.

## Materials and Methods

### Experimental Animals and Ethics Statement

Adult (8–10 weeks old) inbred Fisher F344 rats (Scanbur BK AB, Sweden) were used throughout this study. The animal husbandry was in accordance to the standards and regulations provided by the National Institutes of Health Guide for Care and Use of Laboratory Animals (NIH Publications No. 86–23, revised 1985) and the European Communities Council Directive (86/609/EEC). All procedures were approved by the Northern Swedish Regional Committee for Ethics in Animal Experiments. Surgery was performed aseptically under general anesthesia using a mixture of Ketamine (Ketalar 50 mg/ml Pfizer, Sweden) and Xylazine (Rompun 20 mg/ml Bayer Health Care, Germany) by intraperitoneal injection. Benzylpenicillin (Boehringer Ingelheim; 60 mg) was administered postoperatively. Rats and their wellbeing were observed throughout the experimental period. At the end of the survival period the rats were terminally injected with an intraperitoneal overdose of sodium pentobarbital (240 mg/kg, Apoteksbolaget, Sweden).

### Experimental groups

A sciatic nerve injury was used (see below) and rats were divided into five groups in order to evaluate axonal regeneration, changes in the distal nerve stump and muscle recovery and reinnervation after either immediate (n = 5) or delayed repair at 1 month (n = 5), 3 months (n = 5), and 6 months (n = 4). A separate series of animals was used for retrograde labeling experiments.

### Nerve capping and delayed repair

The sciatic nerve was exposed by bluntly dividing the gluteal muscles of the thigh. The nerve was transected at a standardized distance from the spinal cord at the level of the upper border of the femoral quadrant muscle. In experimental groups with delayed nerve repair, the nerve stumps were ligated approximately 1 mm from the cut end using a 8–0 nonresorbable Ethilon suture to ensure the prevention of reinnervation. Caps were made out of polyethylene tubes. Each stump was introduced and anchored to the bottom of the cap using the 8–0 Ethilon suture [24]. The proximal stump was put under the femoral quad muscle and the distal stump introduced into the popliteal fossa. The wound was closed in layers; muscles with a 6–0 resorbable Vicryl suture and the skin with a 3–0 Silk suture. After 1,3,or 6 months, nerve repairs were performed under an operating microscope (Zeiss, Carl Zeiss, Germany) using micro instruments and a 10–0 nonresorbable Ethilon suture [5]. The proximal and distal stumps of the sciatic nerve were re-exposed, trimmed by 2–3 mm to remove neuroma/scar tissueand repaired using a 10 mm sciatic reversed sciatic nerve graft from a donor Fisher rat. The graft was fixed with four interrupted epineurial sutures aligned circumferentially in each anastomosis and the wound was closed in layers. In the experimental group with immediate nerve repair, the nerve stumps were bridged with a 10 mm sciatic reversed autograft.

### Retrograde labeling of spinal motoneurons

Retrograde labeling of spinal motoneurons regenerating into the distal nerve stump was performed 12 weeks following nerve grafting. The sciatic nerve was transected in the popliteal fossa 10 mm distal to the repair site and introduced into a small polyethylene tube containing two microlitres of fluorescent tracer Fluoro-Ruby (FR, 10% solution in saline, Invitrogen, Sweden). The tube was fixed to the surrounding muscles using Histoacryl® glue (Braun Surgical GmbH, Germany) and sealed with a mixture of silicone grease and vaseline to prevent leakage. Two hours later the cup was removed, the nerve rinsed in saline and the wound closed in layers. The animals were left to survive for one week before tissue harvest.

### Tissue processing

Following immediate and delayed nerve repairs, all animals were allowed to survive for an additional 13-weeks before being given an overdose of sodium penthobarbital (240 mg/kg, Apoteksbolaget, Sweden). The sciatic nerve was removed in its entirety and then three 3 mm pieces of the nerve were cut; 5 mm from the proximal anastomosis, in the middle of the nerve graft and 5 mm from the distal anastomosis. The nerve segments were fixed in 3% glutaraldehyde, post-fixed in 1% osmium tetroxide (OsO4) in 0.1 M cacodylate buffer (pHZ7.4), dehydrated in acetone and embedded in Vestopal. The remaining 5 mm nerve segments immediately proximal or distal to the nerve graft interface were each divided in half and one portion used for Schwann cell in vitro cultures or snap frozen in liquid nitrogen for subsequent RT-PCR analysis. The medial gastrocnemius muscles were harvested, weighed and fast frozen in liquid nitrogen. The other animals labeled with Fluoro-Ruby were transcardially perfused with Tyrode's solution (37°C) followed by 4% (w/v) paraformaldehyde (PFA, pH 7.4). Spinal cord segments L4–L6 were harvested and post-fixed in 4% PFA overnight. The spinal cord segments were cut in serial 50 µm thick parasagittal sections on a vibratome (Leica Instruments, Germany), mounted onto gelatin-coated slides and coverslipped with DPX.

### Neuronal counts

Nuclear profiles of labeled motoneurons were counted in all sections at ×250 magnification under a Leitz Aristoplan fluorescence microscope using filter block N2.1. The total number of nuclear profiles was not corrected for split nuclei, since the nuclear diameters were small in comparison with the section thickness used. We have previously demonstrated that the accuracy of this technique in estimation of retrograde cell death in spinal cord is similar to that obtained with the physical dissector method [7] and counts of neurons reconstructed from serial sections [4]. Preparations were photographed with a Nikon DXM1200 digital camera. The captured images were resized, grouped into a single canvas and labeled using Adobe Photoshop CS4 software. The contrast and brightness were adjusted to provide optimal clarity.

### Axonal count and area assessment

Semi-thin transverse sections of proximal, mid-graft and distal nerve segments were cut on a 2128 Ultratome (LKB, Sweden) and counterstained with Toluidine Blue. Myelinated axons in the proximal and distal nerve stumps, and in the middle of the nerve graft were counted at ×1000 final magnification using the fractionator probe in Stereo Investigator™ 6 software (MicroBrightField, Inc.,USA). The axonal area was calculated by the software after manually marking the outer border of single axons. The area was then described as a mean figure after measuring and summarizing the area of 30 axons at four random sites per cross section and dividing the total area with the number of axons counted.

### RT-PCR

Total RNA was isolated from distal nerve segments and muscle using an RNeasy™ kit (Qiagen, Sweden) and then 1 ng RNA was incorporated into the One-Step RT-PCR kit (Qiagen) per reaction mix. A thermocycler (Biometra, Germany) was used with the following parameters: a reverse transcription step (50°C, 30 min), a nucleic acid denaturation/reverse transcriptase inactivation step (95°C, 15 min) followed by 35 cycles of denaturation (95°C, 30 sec), annealing (30 sec) and primer extension (72°C, 1 min) followed by final extension incubation (72°C, 5 min). Forward and reverse primer (all 5′→3′) pairs (Sigma-Aldrich, UK) with annealing temperatures used are described in table 1. PCR amplicons were electrophoresed (50 V, 90 min) through a 1.5% (w/v) agarose gel and the size of the PCR products estimated using Hyperladder IV (Bioline, UK). Samples were visualised under UV illumination following GelRed™ nucleic acid stain (Bio Nuclear, Sweden) incorporation into the agarose.

**Table 1:** Primer sequences for RT-PCR and annealing temperatures (°C).

Factor	Forward Primer (5′→3′)	Reverse Primer (5′→3′)	°C
S100B	GTTGCCCTCATTGATGTCTTC	AGACGAAGGCCATAAACTCCT	57.9
erbB2	AACCTTTCCTTGCTGCTTGA	GTTCCCTCCAGACCTCTTCC	59.9
erbB3	AGAGGCTTGTCTGGATTCT	AGGAGTAAGCAGGCTGTGT	55.9
erbB4	AACCAGCACCATACCAGAGG	TTCATCCAGTTCTGCTCGTG	62.1
TGF-β	CTAATGGTGGACCGCAACAAC	CGGTTCATGTCATGGATGGTG	67.8
tenascin C	GCCTCAACAACTGCTACAATCGTG	TCAGCCCCTGTGAACCCATC	66.1
collagen I	GTGAACCTGGCAAACAAGGT	CTGGAGACCAGAGAAGCCAC	64.1
phosphacan	GAATTCTGGTCCACCAGCAG	GGTTTATACTGCCCTCTTTAGG	59.0
versican	ACACAGGGAGAAACCCAGGA	TGTCTTGTTTTCTCTGACCT	61.0
α-nAChR	TGTGTCTCATCGGGACGC	GGGCAGAGGGAGGCTTAGTTC	64.0
β-nAChR	CCGTGTCACTGCTGAATCTGT	CTCAAAGGACACCACGACAT	60.9
γ-nAChR	TGTCATCAACATCATCGTCCC	CGAGGAAAAGGAAGACGGT	61.8
δ-nAChR	TGTGGAGAGAAGACCTCG	AGCCTCTTGGAGATAAGCAAC	57.0
ε-nAChR	AACTGTCTGACTGGGTGCGT	GAAGATGAGCGTAGAACCGAC	61.8
MuSK	TGAAGCTGGAAGTGGAGGTTTT	GCAGCGTAGGGTTACAAAGGAA	63.3
18S	TCAACTTTCGATGGTAGTCGC	CCTCCAATGGATCCTCGTTAA	62.1
actin	ACTATCGGCAATGAGCGGTTC	AGAGCCACCAATCCACACAGA	64.1

### Schwann cell (SC) culture

Under a dissecting microsope, the epineurium was removed from the proximal and distal nerve segments. The nerves were further divided into 0.5–1 mm pieces and placed in a petri dish containing Schwann cell growth medium [(Dulbeccos Modified Eagle Medium (DMEM) containing 10% (v/v) foetal calf serum (FCS) and 1% (v/v) pencillin/streptomycin solution (all from Invitrogen) and supplemented with 10 µM forskolin (Sigma) and neuregulin NRG1 (R&D Systems, UK)]. The nerves were incubated for 2 weeks before the addition of 0.0625% (w/v) collagenase type 4 (Worthington Biochemicals, USA) and 0.585 U/mg dispase (Invitrogen) for 24 h. The nerves were triturated, filtered through a 70 µm cell strainer and centrifuged at 800 rpm for 5 min. The pellet was resuspended in 5 ml SC growth media and seeded in a 25 cm^2^ flask. The cells were left to incubate at 37°C/5%CO_2_ for 7 days and then trypsinised and the cell numbers were counted after separation of contaminating fibroblasts using anti-fibroblast antibody coupled magnetic beads according to the manufacturer's instructions (Miltenyi Biotech, Germany). At passage 2, Schwann cells were plated at a density of 7500/well in a 24 well plate and assessed for proliferation over a period of 5 days using the Alamar Blue assay as previously described [25]. Total RNA was also isolated from Schwann cell cultures at passage 2 and RT-PCR performed using the primers described in Table 1.

### Schwann cell-neuron co-culture

Schwann cells (4×10^4^ cells, passage 2) were seeded onto 8 well chamber slides and allowed to settle for 1 day before the addition of 2×10^3^ NG108-15 cells (a neuronal cell line, modeling motor neurons). The two cell types were co-cultured for 48 h and then fixed with 4% (w/v) PFA. Fluorescent immunocytochemistry was used to visualize NG108-15 neurite outgrowth. Briefly, the fixative was removed and the cells washed in PBS and permeabilized using 0.1% (v/v) Triton X-100 for 15 min in the presence of 5% (v/v) normal serum blocking agent. Cells were then incubated with monoclonal βIII-tubulin antibody (dilution 1/500; Sigma, Poole UK) for 2 h at room temperature. Following PBS washes, slides were exposed to secondary goat anti-mouse Alexa Fluor 488 antibody (1∶1000) for 1 h in the dark. After 2 final washes in PBS the slides were mounted with Prolong anti-fade mounting medium containing 4′-6-Diamidino-2-phenylindole (DAPI). The slides were photographed with a Nikon DXM1200 digital camera attached to a Leitz microscope and an average of 150 NG108-15 cell bodies for each condition were analyzed (n = 4 replicates) for neurite outgrowth using Image-Pro Plus software (MediaCybernetics, UK). Neurites were recorded using the trace function. The mean number of neurites per neuron and mean neurite length together with mean length of the longest neurite were calculated.

### Muscle analysis

Sixteen micron transverse sections of gastrocnemius muscles from the contra-lateral and operated sides were cut on a cryostat, fixed with 4% (w/v) PFA for 15 min and then blocked with normal serum. Sections were then incubated with monoclonal primary antibodies raised against fast and slow myosin heavy chain protein (NCL-MHCf and NCL-MHCs, Novocastra, UK both 1∶20 dilution) for 2 h at room temperature. Each slide was also co-incubated with rabbit anti-laminin antibody (Sigma; 1∶200 dilution). After rinsing in phosphate-buffered solution, secondary goat anti-rabbit and goat anti-mouse antibodies Alexa Fluor 488 and Alexa Fluor 568 (1∶200; Invitrogen) were applied for 1 h at room temperature in the dark. The slides were cover-slipped with Prolong anti-fade mounting medium containing DAPI. The staining specificity was confirmed by omission of primary antibodies. Preparations were photographed with a Nikon DXM1200 digital camera attached to a Leitz microscope. Morphometric analysis of muscle sections was performed on coded slides without knowledge of their source. Five random fields were chosen (using the ×16 objective) and images for the immunolocalisation of each myosin heavy chain type plus that for laminin were captured using the appropriate emission filters, and combined to provide dual-labelled images. Each image contained at least 25 individual muscle fibers for analysis. Image-Pro Plus software was calibrated to calculate the mean area and diameter (in µm) for each muscle. The injured side was expressed relative to the contra-lateral control side and the relative mean %±SEM calculated for each group.

For the staining of neuromuscular junctions, antibody against the pre-synaptic marker SV2A was used together with alpha-bungarotoxin (α-BTX) to stain the post-synaptic acetylcholine receptors (AChRs). The muscle sections were fixed and blocked with serum as above and then incubated with polyclonal anti-rabbit SV2A (1∶50, Abcam, UK) for 2 h at room temperature. Following washing, the slides were then incubated for 1 h in the dark with secondary antibody Alexa Fluor 568 goat anti-rabbit IgG (1∶100, Invitrogen) and α-bungarotoxin-FITC (1∶10, Sigma). After washing with PBS, the slides were mounted with Prolong reagent and the sections examined under a fluorescence microscope.

### Statistical analysis

In order to determine the statistical difference between the groups one-way analysis of variance (ANOVA) complemented by Newman-Keuls test (Prism Graph-Pad software) was used. Statistical significance was set as *p<0.05, **p<0.01, ***p<0.001.

## Results

### Regeneration of spinal motoneurons, axonal number and area

**Figure 1:**
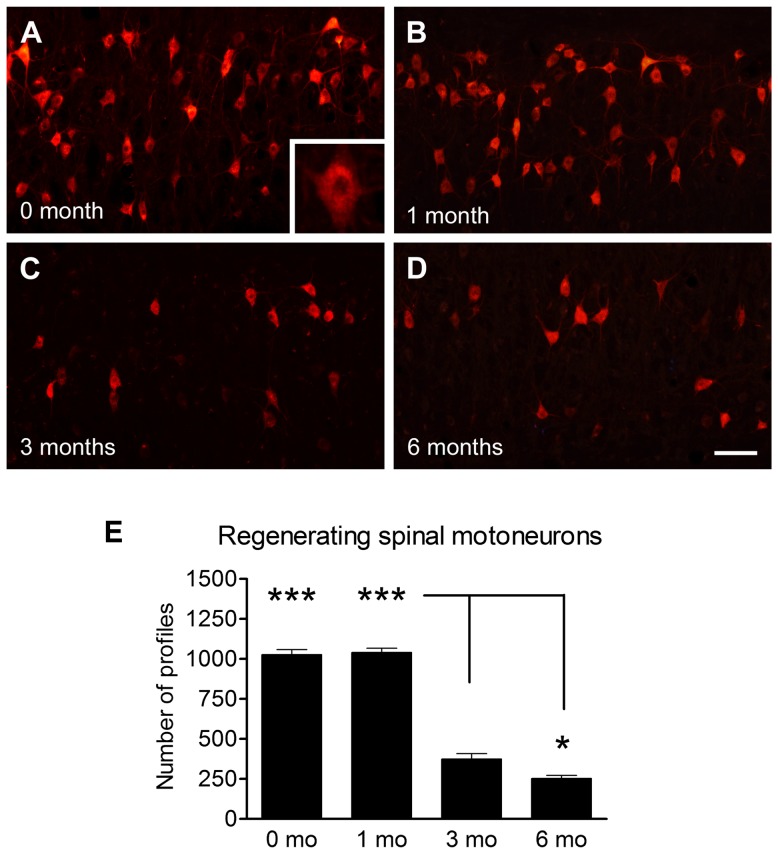
Fluoro-Ruby-labeled motoneurons at 13 weeks after immediate nerve repair (A), 1 months delayed repair (B), 3 months delayed repair (C) and 6 months delayed repair (D). Note significant decrease in number of regenerating motoneurons after 3 and 6 months delayed nerve repair. Scale bar = 100 µm. (E) Histogram showing the quantification of mean±SEM number of regenerating spinal motoneurons after 0–6 months (mo) delayed repair. n = 5, *p<0.05 (3 mo vs, 6 mo), ***p<0.001 (0 mo and 1 mo vs 3 mo and 6 mo).

**Figure 2:**
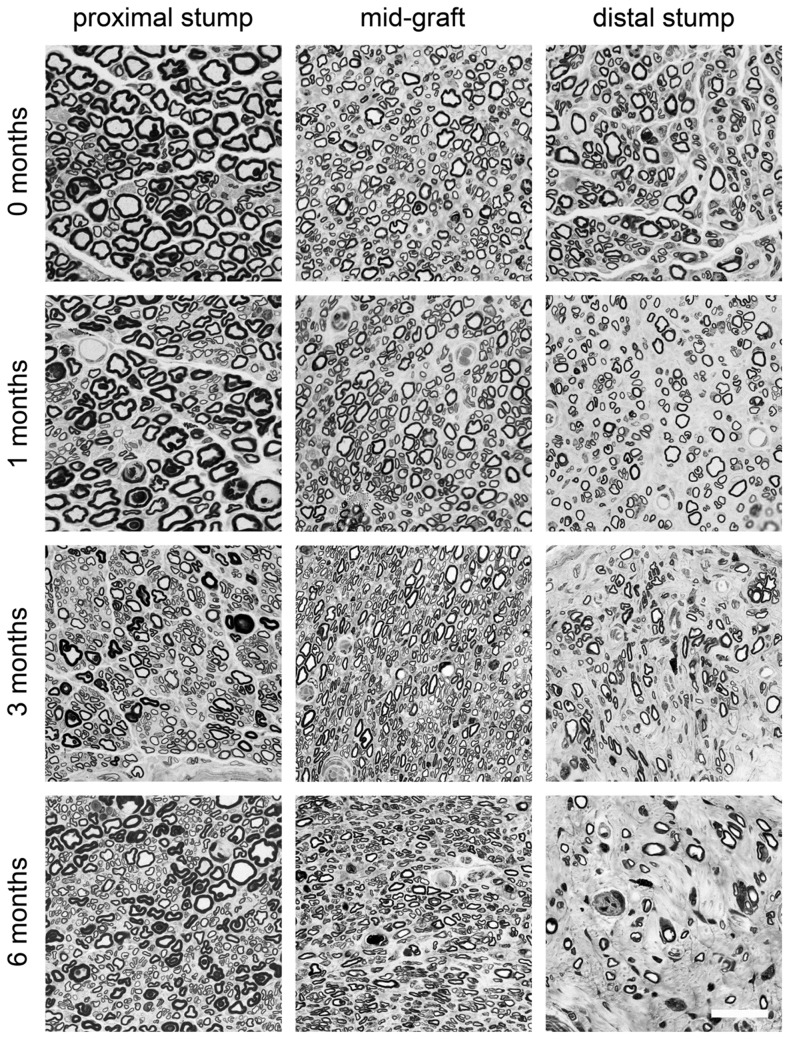
Toluidine blue stained myelinated axons in the proximal stumps, mid-graft regions and distal stumps of animals undergoing sciatic nerve injury followed by either immediate or delayed nerve repair (at the time points indicated) with a 10 **mm graft.** Note increase of number of small myelinated fibers in proximal stump in 3 months and 6 months groups. Scale bar = 30 µm.

**Figure 3:**
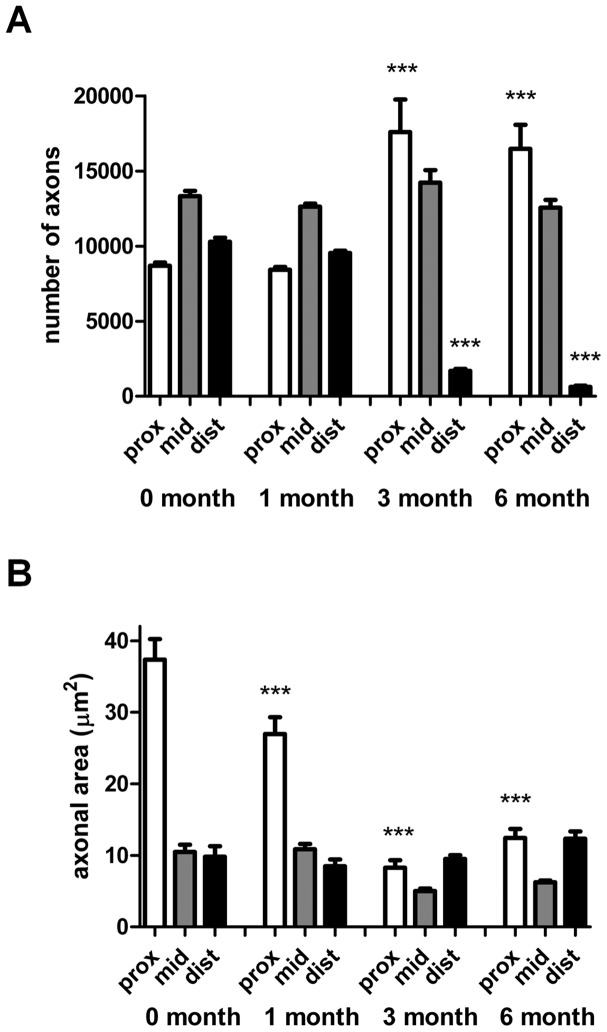
Quantitative analysis of (A) number of myelinated axons and (B) axonal area in the proximal stumps, mid-graft regions and distal stumps of animals undergoing sciatic nerve injury followed by either immediate or delayed nerve repair (at the time points indicated) with a 10 **mm graft.** Values shown are mean±SEM, n = 5, ***p<0.001 significantly different from respective region after immediate nerve repair.

Animals underwent sciatic nerve transection followed by either immediate repair or delayed repair (at 1, 3, or 6 months) using 10 mm nerve grafts and then 13 weeks later motoneuron regeneration was analysed. Segments of nerve were also removed at the proximal, mid-graft and distal regions and analyzed by light microscopy for axonal morphometry. Spinal motoneurons which regenerated their axons across the nerve graft were identified and counted after labeling with a fluorescent dye, Fluoro-Ruby (Figure 1A–D). Following immediate nerve repair or delayed repair for 1 month, 1027±31 and 1041±26 motoneurons had regenerated into the distal nerve stump (Figure 1E). In contrast, delayed 3 months and 6 months nerve grafting drastically reduced the number of regenerating spinal motoneurons to 374±34 and 253±19, respectively (Figure 1E). The proximal stump of nerves from animals undergoing immediate repair showed large diameter axons and sections from the 1 month delayed repair animals appeared similar (Figure 2). In contrast, the 3 and 6 month delayed repair sections showed more numerous axons which were smaller in size (Figure 2). The distal stumps showed a progressive reduction in the number of axons from immediate repair through to the 6 month delay repair animals (Figure 2). Sections from the mid-point of the graft appeared similar throughout all time points (Figure 2). These observations were quantified using Stereo Investigator software (Figure 3). The axonal number in the middle of the graft showed no significant difference between any of the groups. In the proximal segment no difference in the number of axons was observed between the immediate repair and 1-month groups. At 3 and 6 months however the number of axons showed a significant 2-3-fold increase (Figure 3A). In the distal segment the number of axons showed a minimal decline after 1 month delayed repair compared with immediate repair. However by 3 months delay the number of distal axons was significantly reduced by approximately 80–90% compared with both the immediate repair and 1-month groups (Figure 3A). The axonal numbers were further statistically reduced between the 3- and 6-months groups.

The axonal area in the distal stump showed no statistical difference between any of the groups (Figure 3B). In the middle of the graft a small decline in axon area was observed in the 3 and 6 months delayed groups when compared with the immediate and 1 month delay repair groups. Most interestingly, the proximal stump showed a progressive and statistically significant decrease in axonal area at 3- and 6-months delayed repair (Figure 3B).

### Analysis of the distal nerve stump

**Figure 4:**
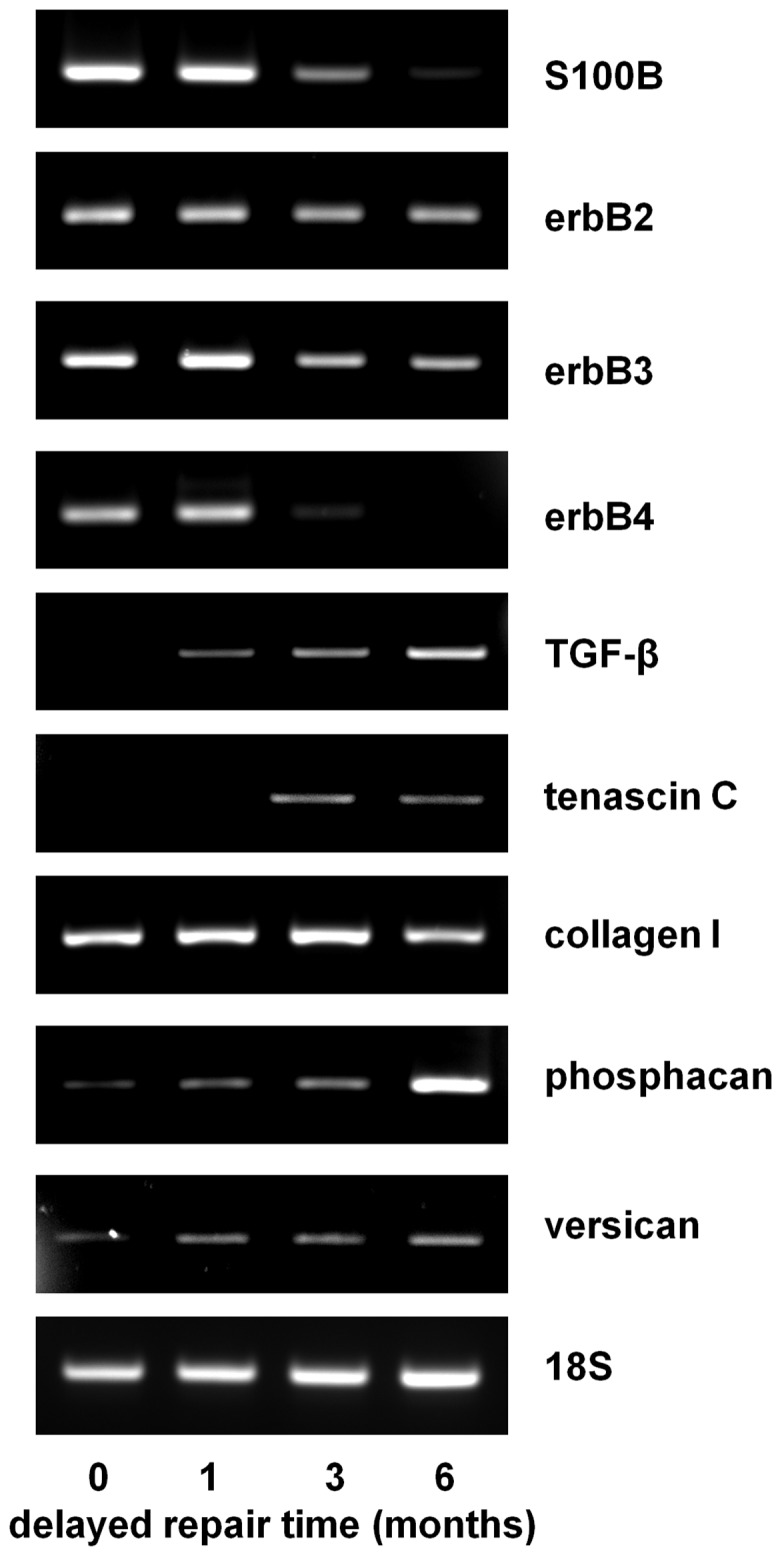
Qualitative RT-PCR analysis of a variety of Schwann cell, axonal and fibrosis/scar associated molecules in the proximal stumps, mid-graft regions and distal stumps of animals undergoing sciatic nerve injury followed by either immediate or delayed nerve repair (at the time points indicated) with a 10 **mm graft.** 18 S is used as a house-keeping gene.

In order to assess the molecular composition of the distal nerve stumps after immediate or delayed nerve repairs, RNA was isolated from the nerve tissue and RT-PCR performed (Figure 4). Compared with the immediate and 1 month delay repair groups there was a noticeable reduction in S100B (Schwann cell marker) mRNA in the 3 and 6 month delay repair groups. The levels of neuregulin/glial growth factor receptors, erbB2-4, were also determined. There was no apparent change in erbB2 expression under the different experimental conditions. In contrast, there was a modest reduction in erbB3 and significant reduction of erbB4 mRNA levels in the distal stumps of animals undergoing delayed repair after 3 and 6 months (Figure 4). A range of fibrotic and scar associated molecules were also assessed. Transforming growth factor beta (TGF-β) was barely detectable in the immediate repair groups but was progressively up-regulated with increase in the time of delayed repair. Similarly, tenascin C expression was only observed in the 3 and 6 month delayed repair animals (Figure 4). Collagen I mRNA was expressed in all distal stumps as was the extracellular matrix proteoglycan versican. Another chondroitin sulphate proteoglycan (CSPG), phosphacan, was only significantly elevated in the 6 month delayed repair group (Figure 4).

### Analysis of the Schwann cells

**Figure 5:**
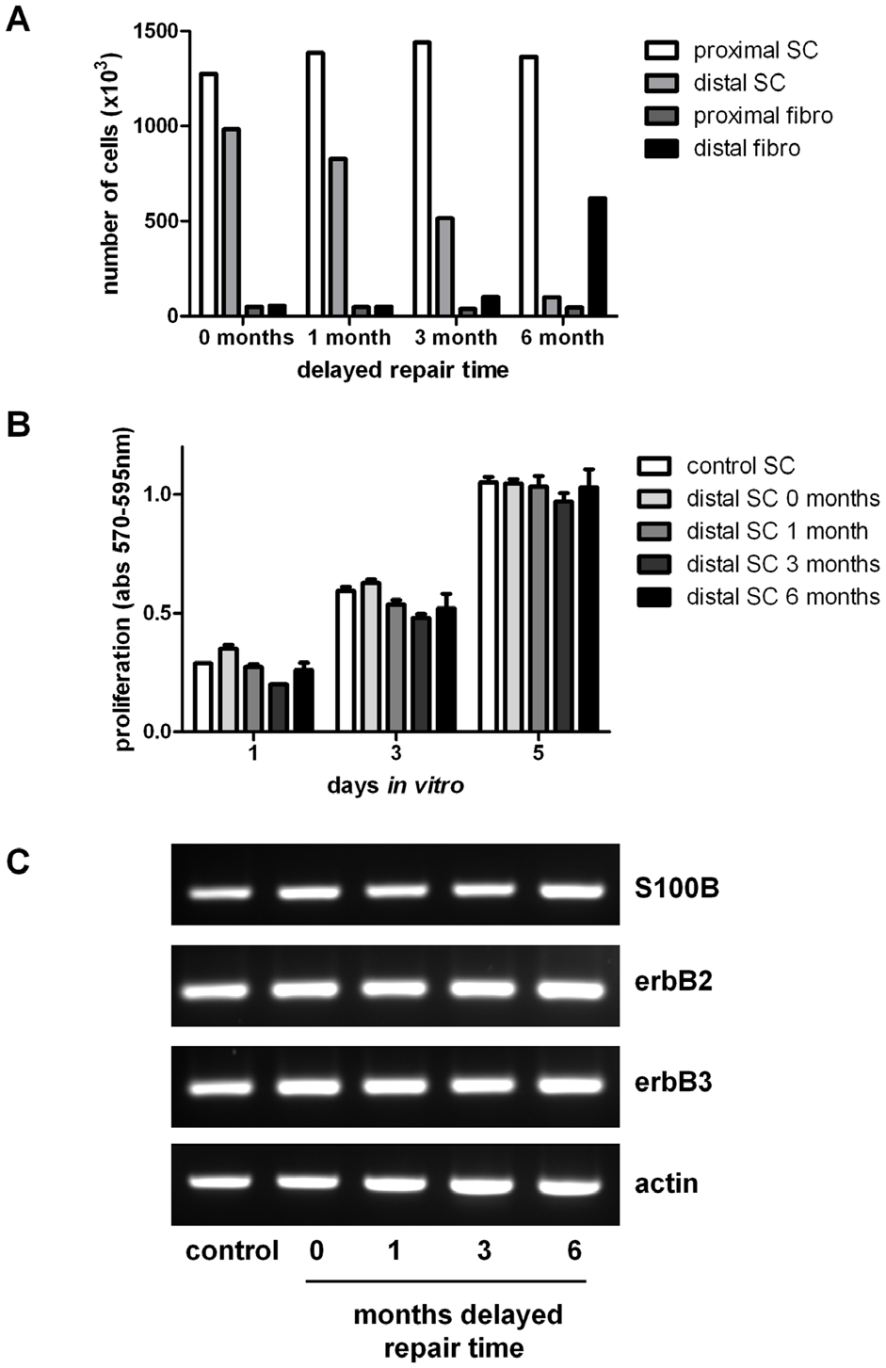
Characterisation of Schwann cells isolated from nerve segments. (A) The number of Schwann cells from proximal and distal nerve stumps of animals undergoing sciatic nerve injury followed by either immediate or delayed nerve repair (at the time points indicated) with a 10 mm graft were counted 7 days following enzyme digestion of the nerve. Fibroblast counts were also made following treatment of the samples with magnetic anti-fibroblast beads. (B) Distal nerve segment Schwann cells from (A) were trypsinised and replated and proliferation rates (in the presence of glial growth factors) compared with control normal cultures of Schwann cells (no experimental injury or repair). (C) Qualitative RT-PCR analysis of Schwann cell marker S100 and the glial growth factor receptors erbB2 and erbB3 expression levels in cultured cells isolated from control nerve (no experimental injury or repair) and the distal nerve segments of animals undergoing immediate (0 months) or delayed nerve repair (1, 3, 6 months). Actin was used as a house-keeping gene.

**Figure 6:**
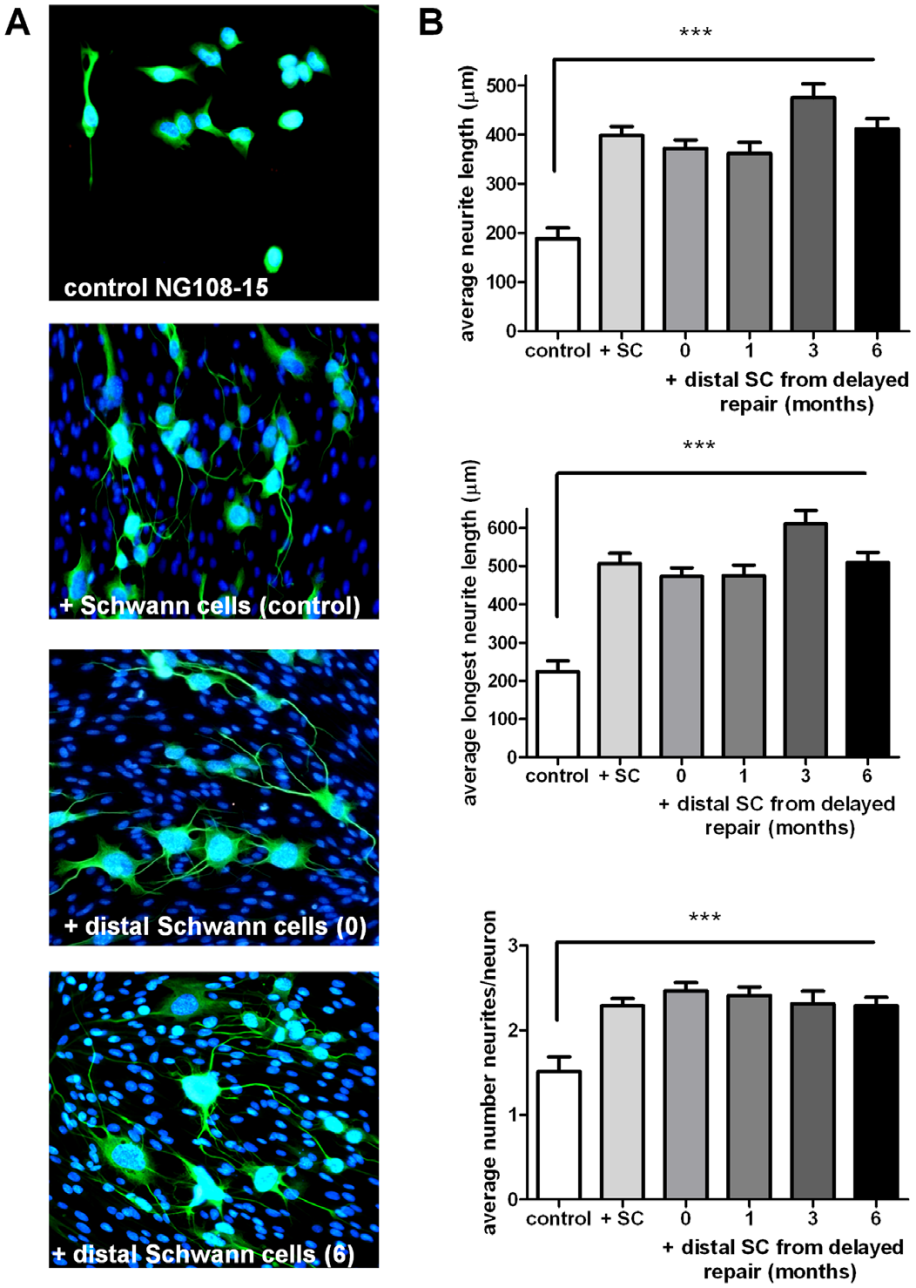
Co-culture of Schwann cells with NG108-15 neurons. (A) NG108-15 cells were either grown alone or on top of a monolayer of Schwann cells isolated from control nerve or distal nerve segments taken from animals undergoing immediate (0) or 6 month delayed nerve repair (6). Cultures were stained with βIII tubulin antibody (green) and DAPI (blue). (B) Quantitative analysis of neurite outgrowth was performed on NG108-15 neurons grown in the absence (control) or presence of Schwann cells isolated from control nerve (+SC) or distal nerve segments from animals undergoing nerve repair immediately following injury (0) or delayed repair at 1, 3, 6 months. ***P<0.001 significantly different from values in the absence of Schwann cells. There was no significant difference between any of the cultures in the presence of Schwann cells.

Schwann cells were isolated from the proximal and distal nerve stumps and expanded in vitro (Figure 5). In all experimental groups a consistent number of Schwann cells could be isolated from the proximal stumps (Figure 5A). In contrast, with a longer delay to nerve repair there was a progressive reduction in the number of Schwann cells which could be isolated from the distal stumps (Figure 5A). This reduction in Schwann cell numbers was paralleled by an increase in the number of contaminating fibroblasts. To determine if these changes were the result of decreased responsiveness to glial growth factors, a proliferation assay was performed (Figure 5B). When cultured in the presence of forskolin and neuregulin, there were no significant differences in the growth rate of Schwann cells isolated from the distal stumps of animals undergoing immediate or delayed nerve repairs. Furthermore, the cells from these animals grew similar to those isolated from control uninjured rats. Consistent with these observations the expression levels of erbB receptors were similar in Schwann cells isolated from all groups (Figure 5C). Next we determined if the Schwann cells isolated from the distal stumps maintained neurotrophic properties. We used our previously published model in which NG108-15 cells (a model motor neuron cell line) are co-cultured on a monolayer of Schwann cells [25]. Schwann cells isolated from uninjured control nerve significantly enhanced neurite outgrowth of the NG108-15 cells (Figure 6). The Schwann cells obtained from the animals undergoing either immediate nerve repair or delayed nerve repair also significantly enhanced the neurite outgrowth and there were no statistically significant differences between groups (Figure 6).

### Muscle analysis

**Figure 7:**
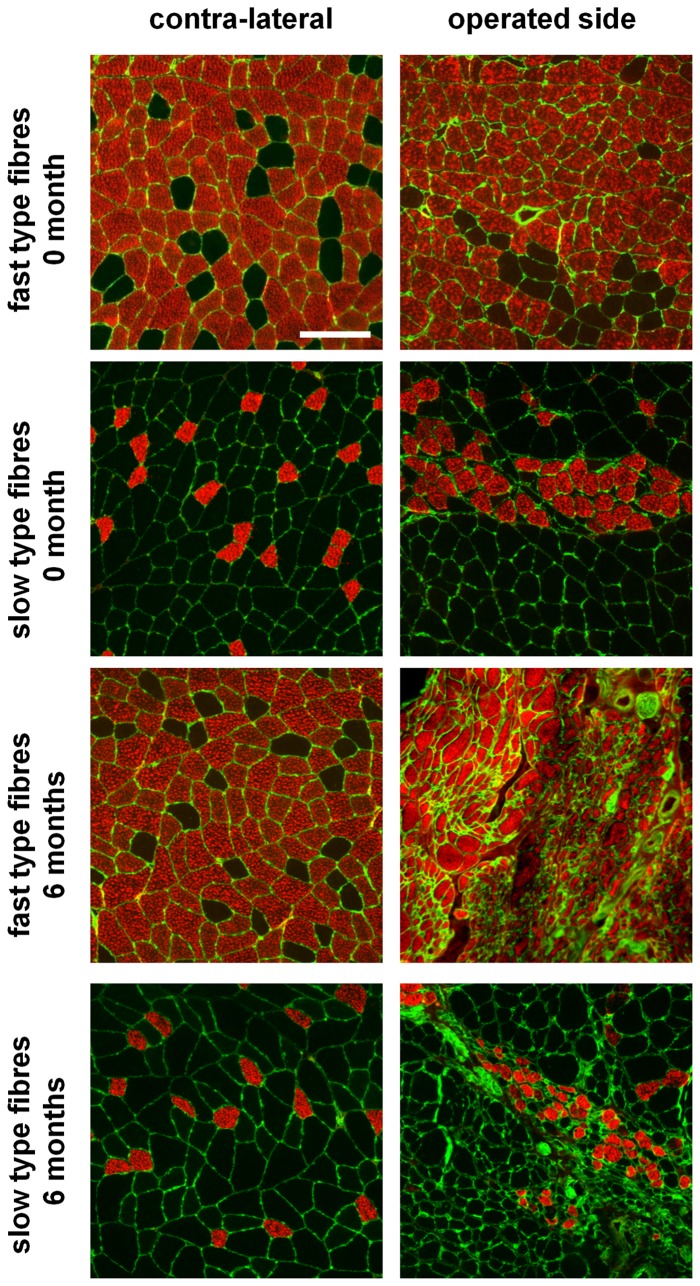
Fast type and slow type gastrocnemius muscle fiber morphology. (A) Transverse sections of contra-lateral and operated side muscles were stained with laminin antibody (green) and either fast type or slow type myosin heavy chain protein antibody (red). Samples shown are from animals undergoing immediate repair (0 months) or delayed repair (6 months). Scale bar = 100 µm.

**Figure 8:**
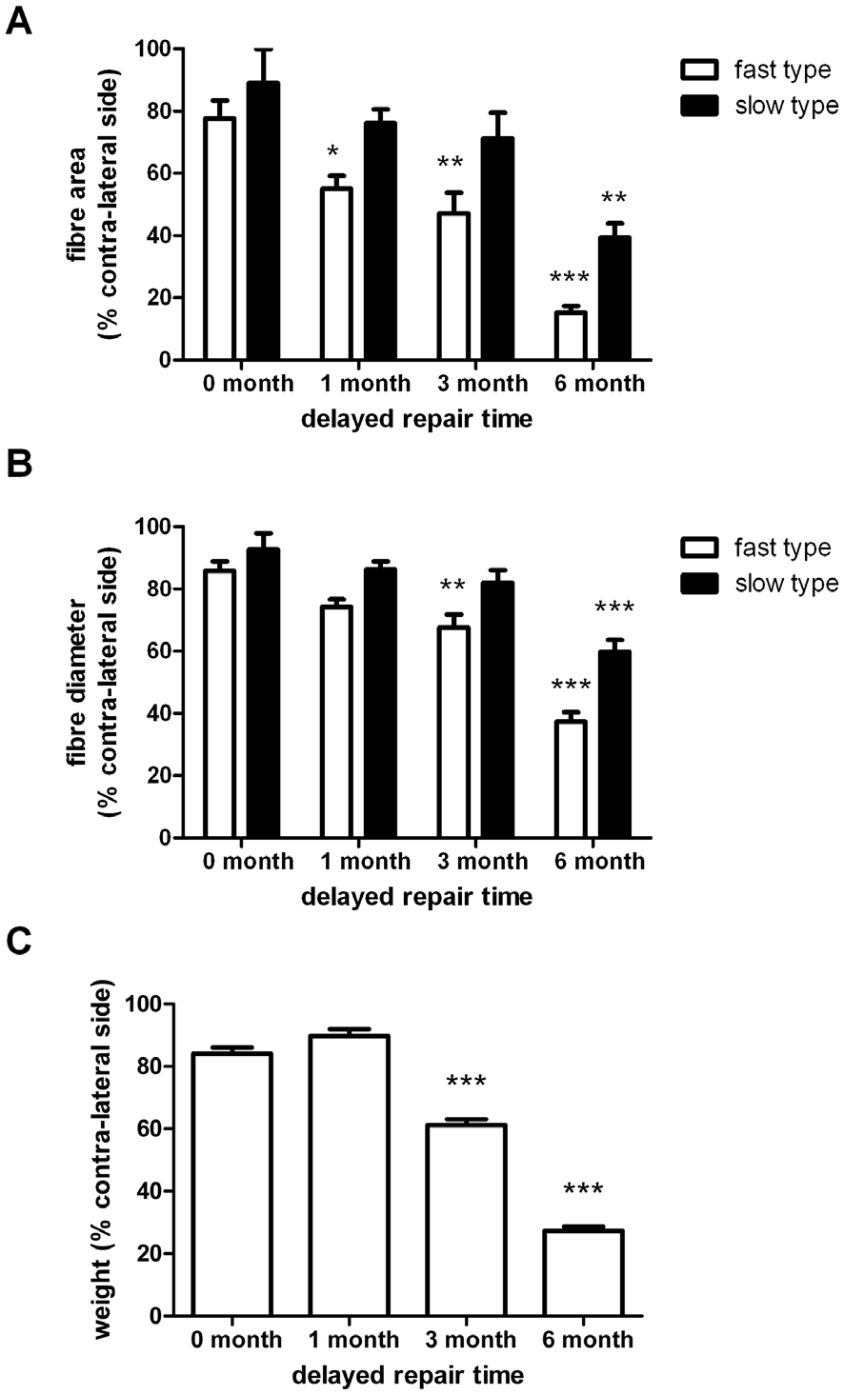
Quantification of muscle fibre size and muscle weights. Computerised image analysis was used to calculate the mean±SEM area (A) and diameter (B) of fast type and slow type fibers in muscle obtained from the contra-lateral and operated sides of animals 3 months after repair. Data are expressed as percentage of the contra-lateral side, *P<0.05, **P<0.01, ***P<0.001 significantly different from respective values in animals undergoing immediate nerve repair (0 months). (C) At the time of harvest, the contra-lateral and operated side muscles were weighed. Data are expressed as percentage of contra-lateral side weights, ***P<0.001 significantly different from weight after immediate nerve repair (0 months).

The medial gastrocnemius muscles from the contra-lateral and operated sides were sectioned and stained with antibodies directed against fast and slow type myosin heavy chain protein (Figure 7). Contra-lateral muscles showed a well organized structure, predominantly populated by fast type muscle fibers. The muscle from the operated side of animals undergoing immediate repair also showed a normal muscle morphology but there was evidence of slow muscle fiber type grouping (Figure 7). In contrast, muscle samples taken from animals undergoing nerve repair after a 6 month delay showed irregular morphology and an apparent reduction in muscle fiber size (Figure 7). Quantitative analysis of the muscle fiber size was performed (Figure 8) and showed a significant reduction in fast type fiber area with 1 month delayed repair and a progressively smaller fiber size in the 3 and 6 month delayed repair animals (Figure 8A). The size of the slow type fibers was not significantly reduced until the 6 month delay repair time point. Similar data were obtained when the fiber diameters were measured (Figure 8B). Animals with immediate nerve repair or after 1 month delay, showed an approximate 20% reduction in the wet weight of the operated side muscles compared with the contra-lateral side (Figure 8C). Consistent with the reductions in muscle fiber size, there was a significant reduction the wet weight of muscles harvested from the 3 and 6 month delayed repair groups (Figure 8C).

**Figure 9:**
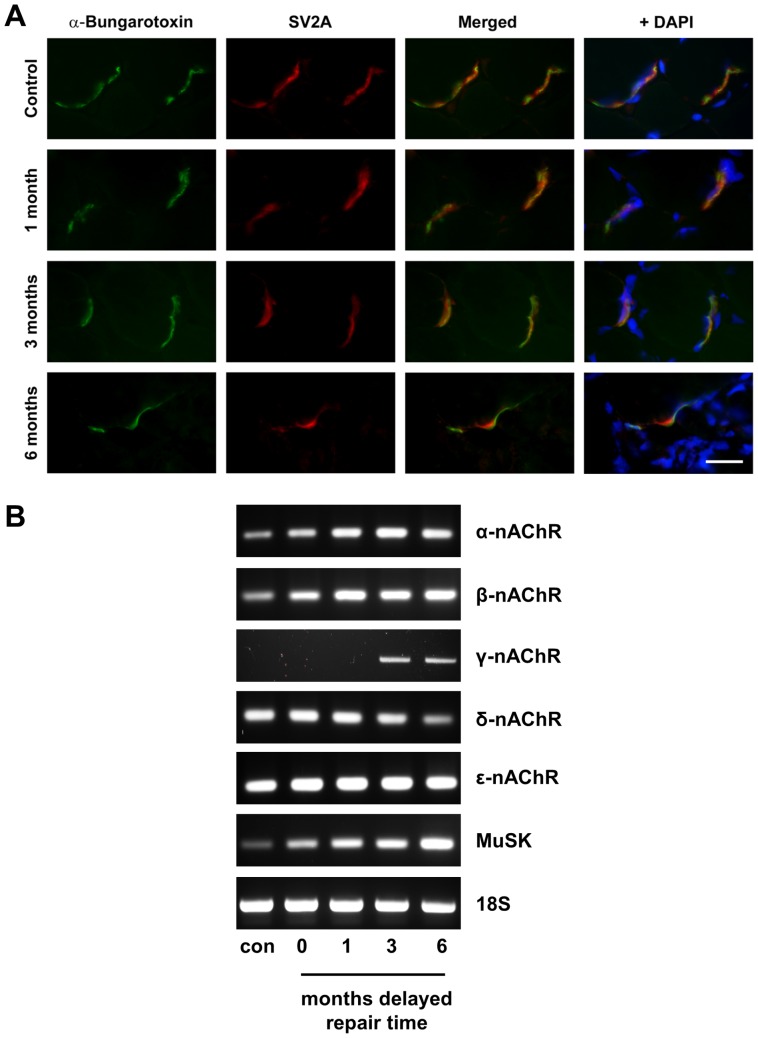
Neuromuscular junctions and expression of nicotinic acetylcholine receptors (nAChRs). (A) Muscle sections were stained for nAChRs with α-bungarotoxin-FITC (green) and with SV2A antibody (red) to mark pre-synaptic structures. Note the coincident staining of both markers indicating presumptive functional NMJs. DAPI staining (blue) shows nuclei. Scale bar = 25 µm. (B) RT-PCR analysis of the different nAChR subunits (α, β, γ, δ, ε) and muscle specific kinase MuSK in control muscle (con) and muscle from the operated side of animals undergoing immediate (0) or 1,3,6 months delayed nerve repair. 18 S was used as a house-keeping gene.

The muscles were also examined immunohistochemically (Figure 9A) using the presynaptic marker, SV2A and α-bungarotoxin (α-BTX) to label the post-synaptic acetylcholine receptors (AChRs). SV2A immunostaining was coincident with the α-BTX binding sites in control muscles. We were able to detect similar neuromuscular junction (NMJ) structures in animals undergoing delayed repair at 1, 3 and 6 months; however at the later time points the NMJs were noticeably sparser. Furthermore, qualitatively the NMJs appeared smaller with increased delay to nerve repair. Changes in motor end plate size and quantity of NMJs are known to be reflected in altered expression levels of genes normally associated with formation of AChRs. To semi-quantitatively measure differences in the NMJs we compared transcript levels of the nicotinic AChR subunits (α, β, γ, δ, and ε) in the muscles following immediate and delayed nerve repair. There were differential changes in expression of the various subunits compared with control muscle (Figure 9B). Of significant note was the detection of the embryonic specific γ subunit in muscle from the animals undergoing 3 and 6 month delayed nerve repairs. The receptor tyrosine kinase, MuSK, an intrinsic protein of the NMJ, is regulated by the innervation state of the muscle. We found that there was a progressively increased expression of the MuSK gene with increase in the delay repair time (Figure 9B). These results further suggest that reinnervation of muscle after prolonged delayed nerve repair is poor.

## Discussion

Our study clearly demonstrates that one of the main limiting factors to target reinnervation and recovery after delayed repair is the degeneration of the distal stump leading to the death of Schwann cells and fibrosis. An equal number of axons are present in the middle of the graft in all groups. However, the number of regenerating spinal motoneurons and myelinated axons projecting into the distal stump follows a steady decline when repair is delayed for more than one month. This results in impaired muscle reinnervation and increased atrophy.

Previous work by Gordon and colleagues has tried to dissect the factors contributing to poor recovery after delayed nerve repair. The role of chronic axotomy [11] or chronic denervation [12] were examined independently. In the first study, the chronically axotomised tibial nerve was used to reinnervate freshly denervated tibialis anterior muscle. It was found that prolonged axotomy reduced the capacity of motor axons to regenerate but did not significantly reduce the number of muscle fibers innervated by each axon [11]. In the second study, the muscle was denervated by cutting the common peroneal nerve before delayed repair with a freshly cut tibial nerve sutured to the distal stump. It was found that progressively fewer axons reinnervated the muscle with prolonged denervation, and this was attributed to deteriorating intramuscular nerve sheaths [12]. Two more studies examining the effect of delayed repair have recently been published which show contradictory results. Kou et al showed that regeneration through 8 week denervated distal nerve was significantly impaired compared with early repair [26]. However, Rönkkö et al., showed that there were no differences in the number of axon sprouts in the distal nerve stump between denervation periods of two and six months [27].

In the present study we used a clinically relevant delayed nerve repair model which allowed us to analyze the combined effects of prolonged axotomy and denervation of the distal nerve stump. The number of axons in the proximal and in the distal nerve stump was approximately equal after either immediate or 1 month delayed nerve repair. However, there were an increased number of axons counted in the nerve graft indicative of sprouting and this number was similar for all time points studied. The mechanism of the sprouting in the middle of the peripheral nerve graft is unclear. Moreover, we have reported previously that only about half of spinal motoneurons and sensory dorsal root ganglion (DRG) neurons are capable of sending their axons from the proximal nerve stump into the distal stump of nerve graft [5], [6].

In addition, we found that there was an increased axonal sprouting in the proximal stump after extended delayed repair. This has been observed previously but no clear conclusions were made [28]. We have previously shown that after 13 weeks of chronic sciatic axotomy only cutaneous sensory DRG neurons undergo significant retrograde cell death whereas the numbers of sensory DRG neurons projecting to muscles and spinal motoneurons remains largely unaffected [5]. More proximal nerve injuries, however, could induce retrograde degeneration of spinal motoneurons [6], [29]. Thus, we hypothesize that the spaces in the proximal stump that were formerly occupied by the axons originating from the cutaneous sensory neurons became replaced by sprouts from spinal motoneurons and muscular sensory DRG neurons. This correlates with the findings of a higher number of axons with a smaller mean diameter in the proximal stump.

The most striking result was the significant decline in the number of regenerating spinal motoneurons and myelinated axons found in the distal stump after 3 and 6 months delay in nerve repair. We used a freshly harvested nerve graft to repair the injury and found that they supported in-growth and sprouting of a similar number of axons at all time points. Our previous studies also demonstrated that injury to the sciatic nerve does not induce any significant loss of spinal motoneurons and does not affect their ability to regenerate into the distal nerve stump [5], [6]. The failure for axons to continue from the graft into the chronically denervated distal stump is therefore most likely to be one the main impediments to reinnervation of peripheral targets. In a more recent study by Gordon et al. it was shown that a chronically denervated nerve graft significantly diminishes the capacity to support regeneration suggesting either decreased neurotrophic support from Schwann cells or an increase in the expression of molecules inhibitory to regeneration [30]. In the same study it was shown that both chronic denervation of the nerve stump together with chronic denervation of the muscle was the greatest impediment to recovery of muscle function [30]. Both of these factors could be considered to contribute to our results.

Our study demonstrated that chronic axotomy significantly decreased the number of Schwann cells in the distal stump but did not affect their neurotrophic and growth-promoting potential in culture. Following axotomy, Schwann cells dedifferentiate and up-regulate various growth factors and extracellular matrix molecules which provide a permissive environment for nerve regeneration. We found that the levels of S100B, a Schwann cell maker, decreased in the distal stump of 3 and 6 months delayed repair groups. This is suggestive that the number of Schwann cells declines with prolonged denervation. Our in vitro findings confirmed the decrease of Schwann cells number which could be isolated from these distal nerve segments. Previous studies have shown that delayed nerve repair increases the number of apoptotic, caspase-3 positive Schwann cells both at the site of lesion and in the distal stump [31]. The same group showed that activating transcription factor 3, a molecule involved in regeneration, was down-regulated in Schwann cells after delayed nerve repair [32]. Schwann cell death occurs under ischemic conditions [33] and it has been shown to be mediated by molecules such as tumor necrosis factor [34] which can act through the low affinity neurotrophin receptor p75 [35]. The Schwann cells we were able to isolate and expand from the longer term denervated distal stumps expressed similar levels of erbB receptors and proliferated in response to neuregulin at similar rates as normal Schwann cells. Previously the levels of erbB2 and erbB4 expression in denervated Schwann cells were shown to be significantly reduced 4 months following denervation and were undetectable in 6 months denervated nerves [36]. The reason we observed high levels of erbB2 in delayed nerve repair cultures could be attributed to the Schwann cells which had migrated from the proximal stump with the few regenerating axons. Nevertheless, our in vivo analysis of the distal segments revealed similar erbB2 expression levels across all time-points studied. However, erbB4 levels were significantly decreased after 3 months delayed nerve repair. Administration of glial growth factor to chronically denervated Schwann cells enhances their proliferation [37] consistent with our in vitro results.

The Schwann cells surviving prolonged denervation could potentially have impaired neurotrophic activity. A study by Hoke et al., showed that a decline in glial cell line derived growth factor expression is associated with impaired axon regeneration after long term Schwann cell denervation [38]. Furthermore, continuous long-term application of exogenous glial cell line derived neurotrophic factor is able to reverse the negative effects of chronic axotomy and significantly enhance motor neuron regeneration [39]. We studied the Schwann cells found in the distal stump after nerve repair. Irrespective of the delay in time to repair, all Schwann cell cultures could support neurite outgrowth of the NG108-15 motor neuron like cell line. This suggests that chronically denervated Schwann cells could be re-activated by regenerating axons [40]. Similarly treatment of chronically denervated nerve grafts with TGF-β and forskolin can enhance regeneration to the distal stump [41]. We cannot however exclude the possibility that the neurotrophic effects we observe in vitro are entirely mediated by Schwann cells migrating from the proximal stump.

In addition to the suggested decline in Schwann cell numbers in the distal stumps of animals undergoing prolonged delay to repair we found a number of inhibitory molecules were up-regulated. TGF-β has been indicated as a factor mediating scar formation and studies have shown that neutralization of this molecule can enhance nerve regeneration [42]. We found increased levels of TGF-β gene expression with delay to nerve repair. However, interestingly we did not find any significant increase in expression of collagen which is known as the main mechanical barrier to regeneration through the scar [43]. Tenascin C, another molecule associated with fibrosis and impaired regeneration in the CNS [44] was also found to be up-regulated after 3 and 6 months delay to nerve repair. Chondroitin sulphate proteoglycans (CSPG), most commonly studied in the CNS glial scar are also up-regulated following peripheral nerve injury [45] but their inhibitory activity is most likely overcome by degradation via matrix metalloproteinases expressed by Schwann cells. Nevertheless peripheral nerve repair can be stimulated by application of chondroitinase enzyme which degrades the CSPG [46]. We found baseline gene expression of both phosphacan and versican in the distal stump of nerve repaired immediately but there was a profound increase in expression of phosphacan and to a lesser extent versican, in the segments from 6 month delayed repair.

We thus found the distal stump showed a decreased capacity to support regeneration after delayed nerve repair, most likely as a result of decreased Schwann cell numbers and increased fibrosis or scarring. These observations were consistent with impaired reinnervation of targets indicated by decreased gastrocnemius muscle weight in the groups subjected to 3 and 6 month delayed nerve repair. In a similar study to ours Kobayashi et al., performed rat tibial nerve transections and either repaired immediately or at delayed time points up to 12 months [47]. In contrast to our results it was shown that axonal regeneration was excellent in all groups, irrespective of denervation period. However, there was a significant decline in muscle mass in groups subjected to delayed repair greater than 1 month, consistent with our data. The changes which occur in the denervated muscle initially involve a myogenic regenerative response to nerve injury followed by progressive atrophy and if denervation is prolonged, subsequent myofiber cell death [48]. In the rat facial muscle it was shown that activated satellite cells could be maintained for up to 8 weeks and faster time to repair was correlated with enhanced numbers of the cells and myogenic response [15]. With prolonged denervation both the satellite cells and mature myofibers undergo apoptosis and muscle tissue is replaced with fibrotic scar and adipose tissue. The rat gastrocnemius muscle has a mixed fiber phenotype and our results show that the fast type fibers are more sensitive to denervation atrophy. Either the slow type fibers have a greater capacity to maintain their size upon denervation or they recover faster/are preferentially reinnervated than the fast fibers. These changes in phenotype will have consequences for the functional output of the muscle. Consistent with the morphological changes of denervated muscle atrophy we also observed changes at the neuromuscular junction level. Destabilisation of NMJs following nerve injury leads to impaired functional recovery. Previous studies have hypothesized that changes in the expression levels of the various nAChR subunits could contribute to this process [49], [50]. In our study, rats undergoing delayed nerve repair at 3 and 6 months showed increased expression of the embryonic specific γ-nAChR subunit suggesting poor reinnervation. The muscle specific receptor tyrosine kinase, MuSK, is an important protein required to coordinate nAChR clustering and differentiation of the NMJ. Previous studies have shown that the levels of MuSK are up-regulated during the aneural stage of muscle regeneration [51], [52]. Compared with control muscle we found that there was a progressively increased expression of MuSK in the muscle with longer delayed nerve repair suggesting that at earlier time-points reinnervation had restored expression levels towards the normal values.

In conclusion our results show that the distal segment of the cut nerve is of critical importance regarding the limitation of nerve regeneration and reinnervation after delayed repair. The “cut-off” point in this experimental rat model seems to be at 3 months, after this point the functional outcome of delayed nerve repair is likely to be limited. It does not seem to be of absolute importance to perform the repair of the nerve immediately though since the results of axonal counts between immediate repairs and 1 month delayed repairs are comparable. However, retrograde neuronal degeneration in DRG is already apparent at 2 weeks after nerve injury [53], [54] and in delayed nerve repairs could lead to decreased sensory recovery. Nevertheless, the data presented in this study will hopefully be able to guide surgeons in deciding timing of nerve repair.
